# Selection of morphological features of pollen grains for chosen tree taxa

**DOI:** 10.1242/bio.031237

**Published:** 2018-04-11

**Authors:** Agnieszka Kubik-Komar, Elżbieta Kubera, Krystyna Piotrowska-Weryszko

**Affiliations:** 1 University of Life Sciences in Lublin, Department of Applied Mathematics and Computer Science, Akademicka 13, 20-950 Lublin, Poland; 2 University of Life Sciences in Lublin, Department of Botany, Akademicka 13, 20-950 Lublin, Poland

**Keywords:** Pollen grains identification, Morphological features, Attribute selection, Classification tree

## Abstract

The basis of aerobiological studies is to monitor airborne pollen concentrations and pollen season timing. This task is performed by appropriately trained staff and is difficult and time consuming. The goal of this research is to select morphological characteristics of grains that are the most discriminative for distinguishing between birch, hazel and alder taxa and are easy to determine automatically from microscope images. This selection is based on the split attributes of the J4.8 classification trees built for different subsets of features. Determining the discriminative features by this method, we provide specific rules for distinguishing between individual taxa, at the same time obtaining a high percentage of correct classification. The most discriminative among the 13 morphological characteristics studied are the following: number of pores, maximum axis, minimum axis, axes difference, maximum oncus width, and number of lateral pores. The classification result of the tree based on this subset is better than the one built on the whole feature set and it is almost 94%. Therefore, selection of attributes before tree building is recommended. The classification results for the features easiest to obtain from the image, i.e. maximum axis, minimum axis, axes difference, and number of lateral pores, are only 2.09 pp lower than those obtained for the complete set, but 3.23 pp lower than the results obtained for the selected most discriminating attributes only.

## Abstract (precis)

**Summary:** We present a novel method for selection of the sufficient pollen grains descriptors set, derived from optical microscopic images, for automatic pollen taxa recognition purposes.

## INTRODUCTION

The basis of aerobiological studies is to monitor airborne pollen in order to determine the abundance of pollen and pollen season timing. This is primarily associated with the continually increasing number of people suffering from airborne allergies (Heinrich, 2002). Recent research shows that in Poland over 45% of its inhabitants suffer from various allergies (Samoliński et al., 2014). The main sources of allergens include pollen grains (Holgate et al., 2001). In the case of Poland, in spring it is birch pollen that shows the strongest allergenic properties (Rapiejko, 2008; Piotrowska and Kubik-Komar, 2012). Hazel (*Corylus*) and alder (*Alnus*) belong to the same family (Betulaceae) and cause allergic cross reactions (Valenta et al., 1991; Rapiejko, 2008). Pollen seasons of the above-mentioned plants partially overlap and thus their pollen grains may be recorded during the same time.

In pollen monitoring, Hirst-type samplers are used, which actively suck in pollen grains from the air (Hirst, 1952). The trapping surface is sticky tape that traps aeroplankton particles. Pollen grains trapped on the tape are examined under a light microscope – they are identified and counted. This task is performed by appropriately trained staff and is difficult and time consuming because the differences in the morphological structure of pollen grains of some taxa are very small and finding them largely depends on the position of the grain on the tape. Moreover, airborne pollen concentrations reach very high values, in particular during the tree pollen period (Piotrowska and Kubik-Komar, 2012), which also translates into slide examination time; for example, during the birch pollen season one microscope slide corresponding to 24 h is examined for 3 h on average. Therefore, there is a need to automate or facilitate this process. In palynological practice, an optical microscope is often the only tool for acquisition of digital images. Therefore, we refer only to the papers devoted to analysis of digital images acquired by optical microscopy. Existing fully automated pollen monitoring systems are largely based on systems other than optical microscopy (Kawashima et al., 2017).

A fully automated taxa classification system should include: (1) microscope slide preparation, including pollen trapping, slide-making capability as well as capability to make images at different focus heights; (2) image recognition; and (3) online presentation of results. The second stage of the system can be divided into two major steps: pollen grain detection and taxa discrimination (Dell'Anna et al., 2010). The first fully automated pollen capture and image-based recognition system was constructed in Germany (Oteros et al., 2015). However, most of the works concerning automated recognition of pollen types propose automation of only some parts of pollen recognition systems i.e. taxa classification (Li and Flenley, 1999) or grain detection and classification from earlier prepared images (France et al., 2000; Chen et al., 2006; Tello-Mijares and Flores, 2016).

The issue that we address in our research is the stage associated with taxa discrimination.

Our aim was to select the morphological features of pollen grains that most strongly discriminate between the three investigated types of pollen (birch, alder and hazel) and at the same time can be easily determined automatically from a microscope image. This selection was made on the basis of the results of classification of the J4.8 decision trees built based on various subsets of features.

When manually classifying pollen grains, a researcher analyzes them under a microscope, paying special attention to the distribution and appearance of the characteristic elements of the grain. The characteristics that distinguish the pollen grain of a specific taxon from other taxa can be the following, among others: grain size and shape, number and arrangement of apertures, exine and intine thickness, exine sculpture, and internal texture.

**Fig. 1.**
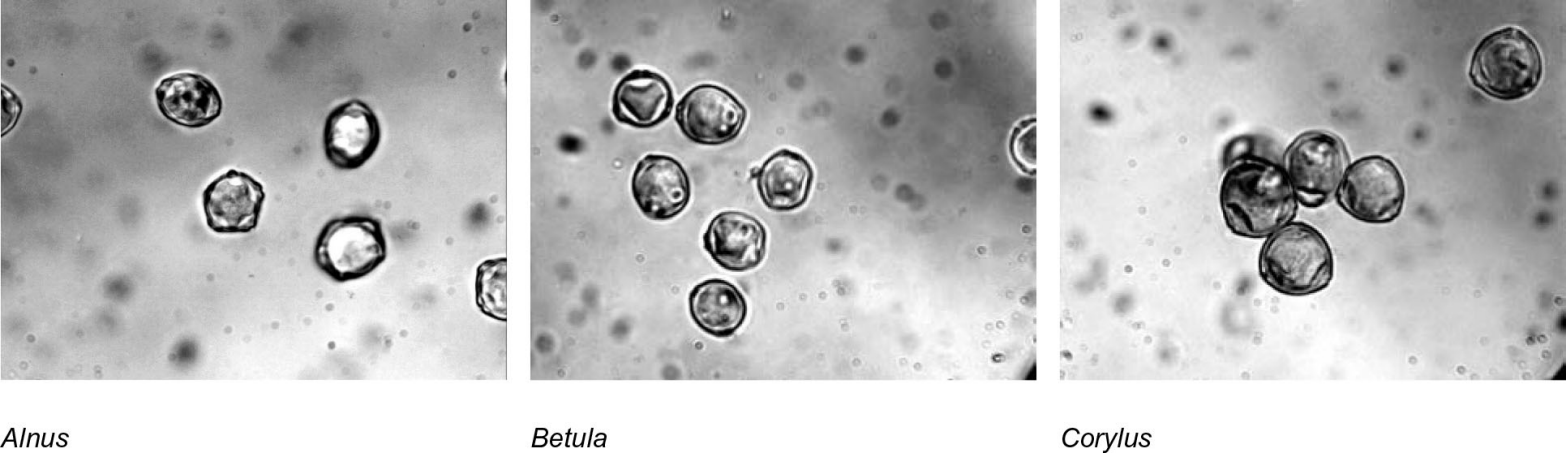
**Examples of microscope images of pollen grains of the studied taxa.** Original microscopic images were converted to grayscale and contrast was stretched for the purpose of readability.

The pollen grains analyzed in this study (examples of their images are shown in Fig. 1) differ from each other primarily in their shape and number of pores; the alder pollen grain has five pores, sometimes four or six, whereas the birch and hazel pollen grains – three (only two are sometimes visible, depending on the position). Unlike the birch pollen grain, the hazel pollen grain is more triangular in polar view and has less protruding pores. The studied pollen grains also differ in oncus size.

Microscopic visibility of pores and onci greatly depends on the position of the grain on the tape. The researcher can control the focus setting of the microscope in order to observe the grain at different depths and thus better perceive the characteristic structural elements. When analyzing a single microscope image, it is much more difficult to correctly describe these characteristics and therefore the procedure of their automated identification should also include automated microscope control and focus adjustment to particular grains. However, it is not the subject of this study.

## RESULTS

**Fig. 2.**
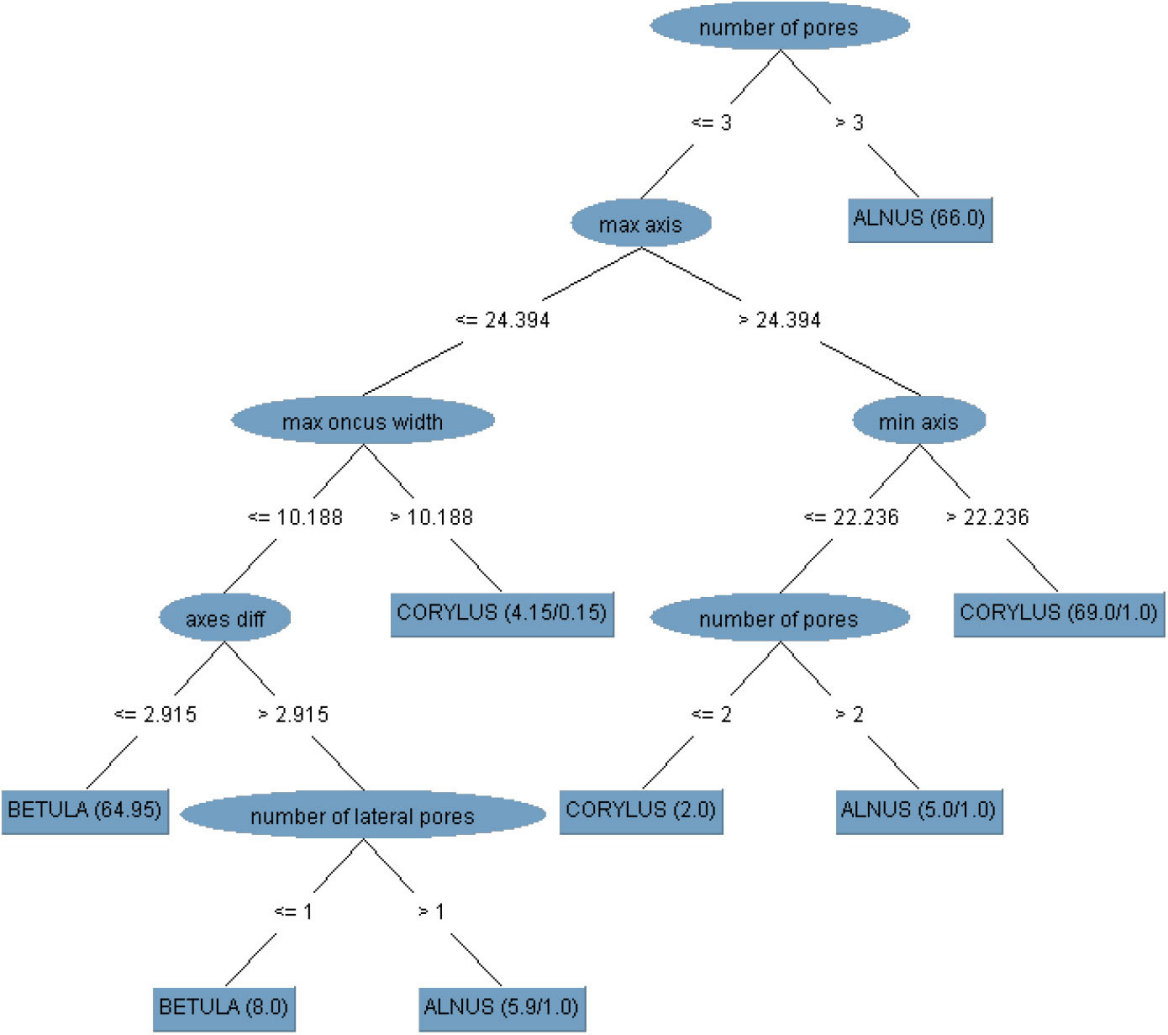
**The J4.8 tree for the full set of features**. The sample size is equal to 225 observations (75 per class indicated by the studied taxa). The training and test sets are the same. The chosen attributes are in elliptical shape, whereas the classes are represented by a rectangle shape. The numbers between the tree branches represent the limit value of the attribute for instance classification. The classification results on the testing set are shown in brackets. The first number in the bracket in the leaf node is the total number of observations (or their weighted number) reaching the leaf. The second number is the measure of misclassification. If the data have missing attribute values and this feature is chosen as the splitting criterion at some level above the leaf node, then these numbers are weighted. min, minimum; max, maximum; diff, difference.

The classification tree was built based on all the features determined and on the full set of observations is shown in Fig. 2. A detailed description of the tree diagram structure created in WEKA is presented in the WEKA documentation (Witten et al., 2016).

The tree diagram shows that the feature that most distinguishes *Alnus* from the other taxa is number of pores, which had a value of over three in most cases for this taxon (66 out of 75). The other grains had three visible pores at the most. Among these observations, it was possible to classify *Corylus* at the lower levels of the tree mainly due to the size of the grain axis. For 69 out of 75 pollen grains of this taxon, the values of the above-mentioned attribute were greater than 24.39 µm for the maximum axis and 22.24 µm for the minimum axis. Fig. 2 shows that *Betula* is the taxon with the smallest grain size and that the maximum axis in most of its grains is not more than 24.39 µm. Furthermore, the other taxa can also have small grains, but the features that additionally distinguish most birch grains are the maximum oncus width of not more than 10.19 µm and a smaller difference between the axes compared to the other taxa.

The percentage of correct classification of observations on which the tree was built was 98.67%.

Table 1 shows the results of the fivefold cross-validation. Apart from the percentage of correct classification, the features included in the nodes of each tree are also specified.

**Table 1.**
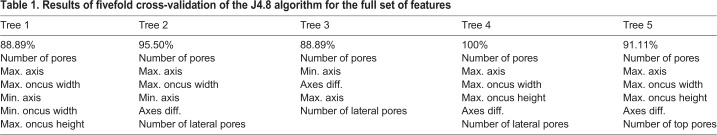
**Results of fivefold cross-validation of the J4.8 algorithm for the full set of features**

The average percentage of correct classification for the fivefold cross-validation was 92.89%. When this procedure was repeated 1000 times, an almost identical result (92.81%) was obtained.

Based on the results presented in Fig. 2 and Table 1, a conclusion has been drawn that the following are the features that most strongly discriminate between the studied taxa: number of pores, maximum axis, minimum axis, axes difference, maximum oncus width, number of lateral pores. Let us denote this set as Best6.

**Fig. 3.**
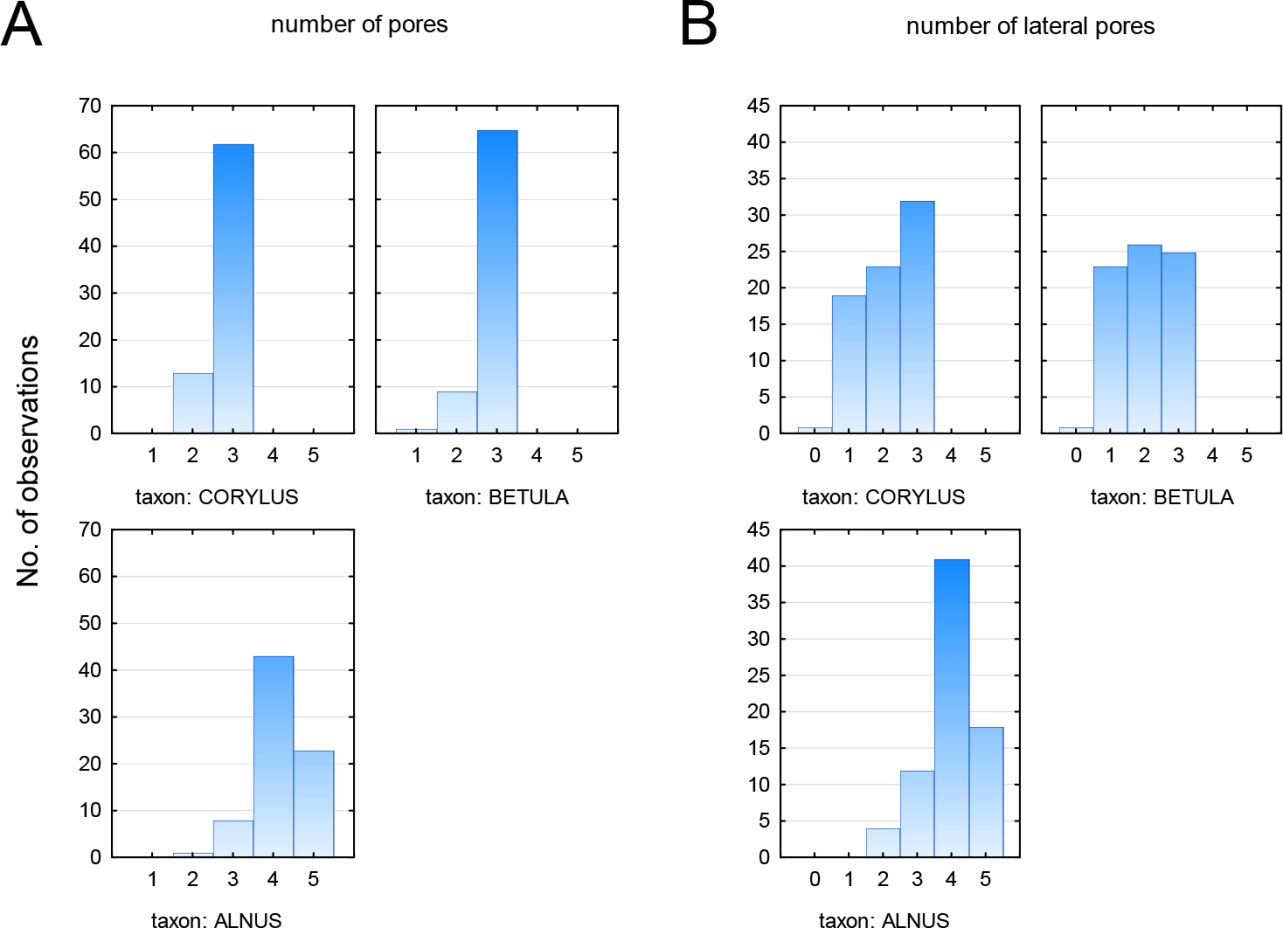
**Histograms of (A) the number of pores and (B) the number of lateral pores for the studied taxa.** The sample size is equal to 75 observations for each taxa.

**Fig. 4.**
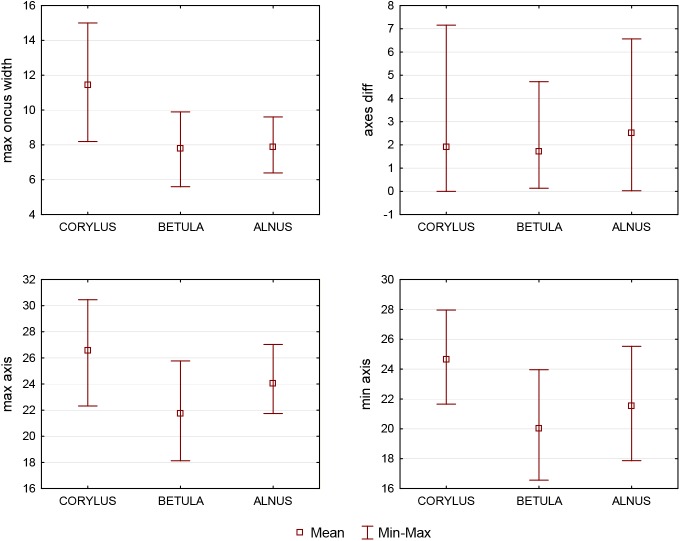
**Plot of the mean value and the min.-max. range of the selected features for the studied taxa.** The square represents the mean value, whereas the length of the whisker determines the feature interval. The sample size is equal to 75 observations for each taxa.

Fig. 3 shows a histogram of two features: number of pores and number of lateral pores, whereas Fig. 4 presents the range of variation of the other features from Best6 for the taxa analyzed. These graphs reflect the situation presented in the tree structure in Fig. 2 and described above. It can be seen that for a large majority of *Alnus* grains the number of pores and the number of lateral pores are above three, which makes these traits good features to distinguish this taxon from the other ones. In turn, it can be seen in Fig. 4 that, except for the differences between the maximum axis and the minimum axis, the average values of the features presented in this graph are higher in the case of *Corylus* relative to the other taxa.

In order to determine number of pores, we must first determine the number of top pores, number of oblique pores, and number of lateral pores. Likewise, to determine maximum oncus width, we must determine the width of all onci. From that we can also easily determine the minimum value of this quantity. Thus, the following features can be added at low cost to the Best6 features: number of top pores and number of oblique pores as well as minimum oncus width. This set of attributes is denoted as Best6+3.

Hence, the next step in simplifying the model was to narrow down the set of features to the above-mentioned groups of attributes and to verify this classification using fivefold cross-validation.

This model validation method alone was sufficient since the tree built on all observations with the narrowed set of features is identical as in Fig. 2.

The results presented in Table 2 show that the classification accuracy of the trees built on the six most strongly discriminative features is higher than for the trees built on the wider set. This may be associated with overlearning, that is, the overfitting of the tree to the training data, but our observations reveal that this is primarily associated with the selection of the best splitting attribute at a particular stage of tree building, even if the division obtained in additional several steps could prove to be better.

**Table 2.**
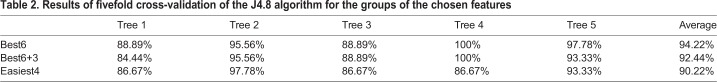
**Results of fivefold cross-validation of the J4.8 algorithm for the groups of the chosen features**

**Fig. 5.**
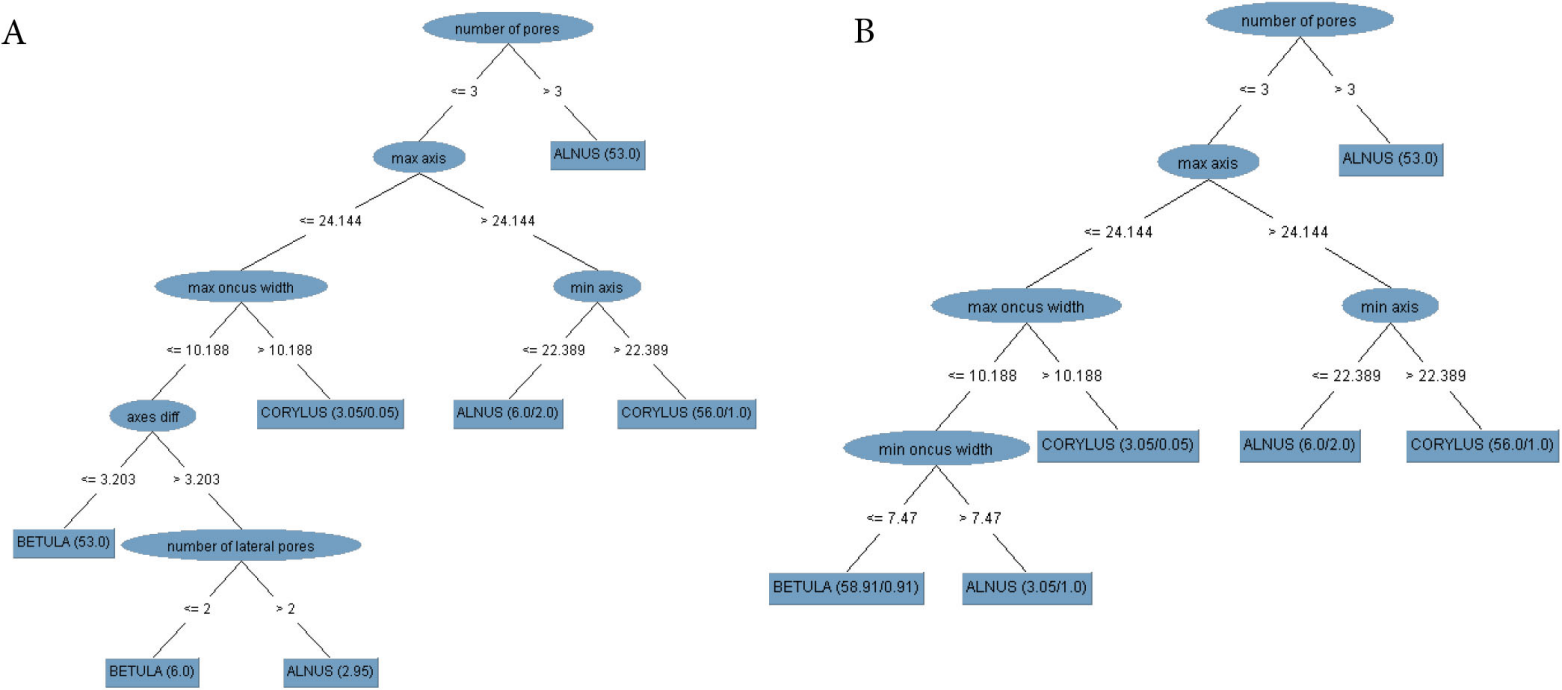
**Structures of Tree 1 for the (A) Best6 and (B) Best6+3 groups.** Best6 consists of the following features: number of pores, max. axis, min. axis, axes diff., max. oncus width, number of lateral pores. Best6+3 consists of Best6 + number of top pores, number of oblique pores and min. oncus width. The sample size for building these trees was equal to 180 observations (60 for each taxon) while the verification set consisted of 45 instances (15 per class indicated by the studied taxa). Cases in the training and test sets did not overlap. The chosen attributes are in elliptical shape, whereas the classes are represented by a rectangle shape. The numbers between the tree branches represent the limit value of the attribute for instance classification. The classification results on the testing set are shown in brackets. The first number in the bracket in the leaf node is the total number of observations (or their weighted number) reaching the leaf. The second number is the measure of misclassification. If the data have missing attribute values and this feature is chosen as the splitting criterion at some level above the leaf node, then these numbers are weighted.

As an example, we present the structure of Tree 1 from Table 2 for both sets of features (Fig. 5).

It can be seen that the tree for the Best6+3 group selected the attribute minimum oncus width, which immediately discriminates between birch and alder at the leaf level, but with worse efficiency in the test than the two-stage division obtained in the tree for the Best6 group using the features axes difference and number of lateral pores.

When the model validation procedure was repeated 1000 times, the classification accuracy was 93.95% for Best6 and 93.12% for Best6+3.

From the point of view of image analysis, determining all the Best6 features can be troublesome. We made an attempt to build classifiers based on the easiest, in our opinion, features to be automatically identified among these six features and these were the following: number of lateral pores, minimum axis, maximum axis, and axes difference. This feature set is denoted as Easiest4.

**Fig. 6.**
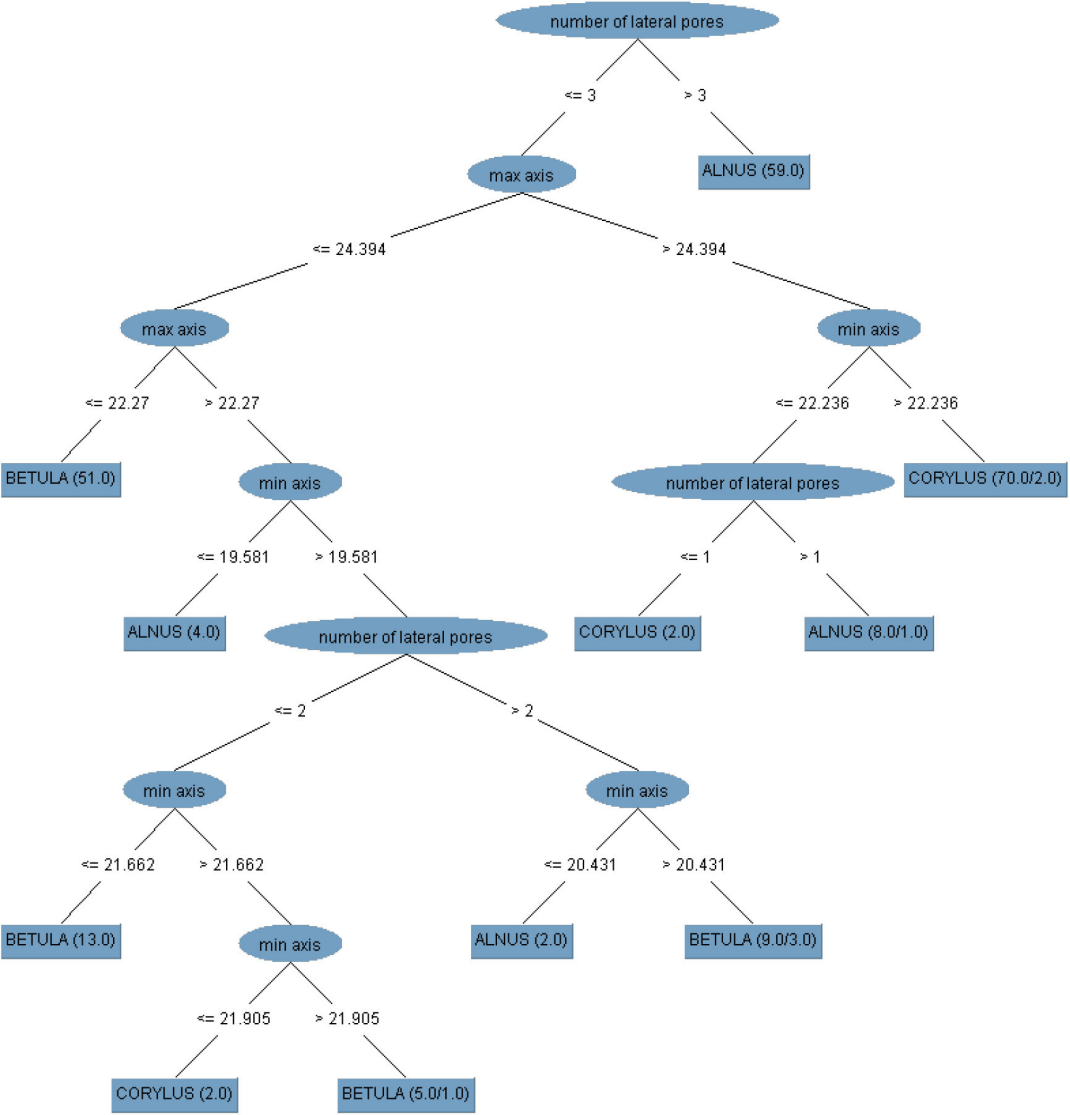
**The J4.8 tree based on the four features easiest for automatic recognition.** These attributes are as follows: number of lateral pores, min. axis, max. axis, and axes diff. The sample size is equal to 225 observations (75 per class indicated by the studied taxa). The training and test sets are the same. The chosen attributes are in elliptical shape, whereas the classes are represented by a rectangle shape. The numbers between the tree branches represent the limit value of the attribute for instance classification. The classification results on the testing set are shown in brackets. The first number in the bracket in the leaf node is the total number of observations (or their weighted number) reaching the leaf. The second number is the measure of misclassification. If the data has missing attribute values and this feature is chosen as the splitting criterion at some level above the leaf node, then these numbers are weighted.

For the training based on all observations, the correct classification of the same set was 96.89% (Fig. 6). The results of the fivefold cross-validation for Easiest4 features set are also presented in Table 2.

It can be seen that number of pores and maximum oncus width, omitted in the construction of the models for Easiest4, were of major importance in the discrimination of taxa. The percentage of correctly classified data decreased by almost 4 pp. However, it is worth noting that the classification accuracy, based on only four features that are relatively easy for automatic identification, is more than 90%. When the construction of the tree based on the four-feature set was repeated 1000 times, the percentage of correct classification was 90.72%.

## DISCUSSION

Our research was focused on the taxa discrimination part of the pollen monitoring system. In most of the studies devoted to this stage, the image-based taxa classification has been carried out using neural networks (Li and Flenley, 1999; France et al., 2000; Tello-Mijares and Flores, 2016). Other types of classifiers have been used less frequently, e.g. the nearest neighbor method (Chen et al., 2006; Bonton et al., 2001; Boucher et al., 2002), bayes classifiers (Ranzato et al., 2007; Tello-Mijares and Flores, 2016), the support vector method (SVM) (Marcos et al., 2015; del Pozo-Baños et al., 2015), and random forest (Tello-Mijares and Flores, 2016).

The selection of the decision tree as the method for obtaining the model in our work was not accidental. This classifier performs well for smaller datasets and besides it provides full information on the classification at each stage of division. One can observe what features are selected at individual tree levels, what the threshold values of these features are, and what subsets are determined by them. Hence, when building a tree model, we obtain an accurate insight into the classification process, which in turn translates into identification of relationships between the features describing the research material and the individual classes that we are considering. We realize that the classifier chosen by us (decision tree) belongs to simple classifiers and the classification quality would certainly have been higher if we had used a different mechanism, but at this stage our aim was to describe in detail the classification rules and the decision tree enables such rules to be inspected, unlike the above-mentioned classifiers.

In our study, we focused on the discrimination of the following taxa: (1) the ones that are common in Poland and exhibit a very strong allergenic effect; (2) with partially overlapping pollen seasons; and (3) the similarity of their pollen grains is high and therefore discrimination between them is problematic.

The studied set of taxa has a significant effect on classification results. The pollen grains of four plant types typical of New Zealand: *Hoheria populnea*, *Phormium tenax*, *Phymatosorus novaezelandiae*, and *Podocarpus totara* can be easily discriminated due to their shape. However, differentiating them only by the texture features allowed 100% of correctly classified instances to be obtained (Li and Flenley, 1999). The first automated system for detection and classification of pollen grains of *Polemonium caeruleum*, *Nymphaea alba*, and *Crataegus monogyna*, successfully obtained the average classification rate at a level of 83% (France et al., 2000). The three above-mentioned species produce pollen grains that clearly differ from one another, in particular in terms of their exine and aperture structure.

A high pollen recognition rate, as much as 96%, was also achieved in the case of automated analysis of pollen of 12 plants typical for Mexico (Tello-Mijares et al., 2016). Most of the investigated pollen grains strongly differ both in shape and in size, but among them there are also grains that are similar to one another in terms of their structure.

Pollen recognition with regard to Cupressaceae, *Olea*, *Parietaria*, Poaceae and taxa similar to them was the subject of research related to the creation of a pollen recognition system under the European ASTHMA project (Bonton et al., 2001; Boucher et al., 2002). Pollen seasons of these taxa partially overlap and therefore their pollen grains can occur in the air at the same time. These are strongly allergenic plants and hence it is important to monitor them. In microscopic analysis, it is possible to confuse the pollen of grasses and that of Cupressace, because in the latter ones the star-shaped outline of the cytoplasm cannot be seen (as is the case in young grains), while in grasses the pore is poorly visible. In microscope slides, three pores are sometimes poorly visible in *Parietaria* pollen grains and they can be confused with monoporate grass pollen grains. Thus, the classification result at a level of 77% achieved in this study seems to be very high.

As we mentioned above, discrimination between taxa with similar features of grains is a very challenging task. An example of such research is pollen recognition for three taxa of the Urticaceae family (Rodriguez-Damian et al., 2006), where the best result of correct pollen recognition was 89%, which is extraordinarily high.

On the other hand, the high result of 97.2% for distinguishing between grass, birch and mugwort pollen grains using an automated system (Chen et al., 2006) is not surprising because these grains significantly differ from one another both in size and shape, as well as in the presence of apertures.

In our research, the best achieved taxa classification accuracy was almost 94%. Nevertheless, it should be remembered that a direct comparison of the percentage values is unreliable, due to the different levels of similarity between the taxa investigated in these studies as well as different classifiers or feature vectors.

The common practice in the above-mentioned studies is to automatically acquire pollen grain attributes from the transform of an original image. In our work, we present a classification based on a small number of features directly measured by the palynologist. It seems that the system based on such features is more intuitive and easier to control. Even though we have chosen a small number of features, the obtained classification accuracy is at a similar level as in the above-mentioned studies.

Despite that classification of pollen grain taxa is not a new subject, our approach to this topic essentially differs from the procedures presented in the literature in this area. In determining the discriminative features, we directly link them to the specific taxa and at the same time obtain a high percentage of correct classification. One of the latest works describing a feature selection before pollen classification is the paper where dimensionality reduction is made using Linear Discriminant Analysis (del Pozo-Baños et al., 2015). However, the attribute selection method, given by the above-mentioned authors, differs from ours, since it generates a space of new synthetic variables with a different interpretation, whereas in our case we leave the subset of the most strongly discriminating original features.

In summary, the approach used here allowed us to present, that among the 13 attributes analyzed, the most strongly discriminating features were the following: number of pores, maximum axis, minimum axis, axes difference, maximum oncus width, and number of lateral pores, and the classification based on this subset was better than that based on the full set. We can therefore conclude that in building classification trees a good practice is earlier selection of attributes due to the possibility of tree overfitting, and the proposed method may lead to better discrimination results in any classification task.

The essential rules resulting from the structure of the classification tree allow us to conclude that the feature that most distinguishes *Alnus* from the other taxa is the number of pores higher than three, whereas *Corylus* grains can be automatically distinguished from the other grains by using the features that determine grain size (greater than 24.39 µm for the maximum axis and 22.24 µm for the minimum axis). Among the small size grains, birch grains are characterized by the maximum oncus width not greater than 10.19 µm and smaller, compared to the other pollen grains, differences between the axes (<2.92 µm).

The classification based on the features easiest to obtain from the image: maximum axis, minimum axis, axes difference, number of lateral pores, allows us to obtain a classification worse by only 2.09 p.p. than that done on the full set of features and by 3.23 p.p. in relation to the selected most discriminative attributes. It is worth indicating that such a small number of features already provides discrimination between these three taxa at a level of more than 90%. Therefore, in our opinion an automated recognition system based on automated identification of these four features can be useful for an expert in pollen monitoring.

These rules may form the basis for discrimination between the studied taxa. Moreover, the proposed attribute selection procedure can be applied to other sets of taxa in pollen recognition systems.

## MATERIALS AND METHODS

### Biological data

In Poland the most frequently occurring species of the studied taxa are *Corylus avellana* L., *Alnus glutinosa* (L.) Gaertn. and *Betula verrucosa* Ehrh (syn. *B. pendula* Roth) (Zając and Zając, 2001).

This study was conducted based on reference slides of pollen grains of the above-mentioned species. The microscope images were analyzed using a Nikon Eclipse E400 biological microscope at a magnification of 600×. A HDCE microscope camera - ×5 - was used for pollen grain measurements.

For the needs of this paper, a base of morphological features was created manually by one of the authors. Values of these features were read under the microscope for 225 pollen grains (75 grains for each of the taxa analyzed). The following attributes were measured and calculated:

(1-2) Equatorial and polar length. The minimum and maximum values were adopted as features: minimum axis, maximum axis;
(3) The difference between the maximum and minimum axis: axes difference;
(4) Exine thickness: wall;
(5-8) Number of visible pores, with distinction between pores visible from above (the position characteristic for pollen grains in equatorial view), laterally situated pores (visible for the grain in polar view), and obliquely situated pores (that is, an intermediate position between the two above-mentioned ones), and also the total number of visible pores: number of top pores, number of lateral pores, number of oblique pores, number of pores;
(9-10) Oncus height. The minimum and maximum height based on measured heights: minimum oncus height, maximum oncus height. In the set, there were observations for which it was not possible to determine any oncus height and then a given observation had missing values for these attributes;
(11-12) Oncus width. The minimum and maximum width based on measured widths: minimum oncus width, maximum oncus width. Similarly as above, there were missing values in the set;
(13) Pollen grain position. The following positions were included: polar: (P), equatorial (E), and three types of intermediate position: intermediate, nearly equatorial and nearly polar: position.

### Decision tree

The decision tree is defined as a graph representing the process of dividing a set of objects into homogeneous classes. This division is based on a set of attributes.

A tree consists of (1) the root, where the full set of instances is taken into consideration, (2) nodes where a decision is made by checking the condition (test) and the current set is divided into subsets, (3) the branch leading to the next level of nodes, and (4) the leaf – a class to which the observation is assigned (Maimon and Rokach, 2010). Thus, instances are classified by navigating them from the root of the tree down to a leaf, according to the outcome of the tests along the path.

The recursive algorithm building a tree which is running on each node is based on the attribute selection criterion for choosing the most appropriate attribute for dividing observations into classes. This procedure gives us information whether the node should be a leaf or a node-branching.

The obtained model can be verified by (a) using the test sample; (b) v-fold cross-validation when a test sample is not available.

The advantages of the decision tree method:

- as a non-parametric method, it does not require information about data distribution;
- it can classify both categorical and numerical data;
- the class of functions describing the effect of independent variables on the dependent variable can be unknown;
- it is resistant to outliers.

Where the dependent variable is a nominal variable, the decision tree becomes a classification tree.

In this study, to discriminate between the analyzed taxa, the J4.8 tree was used due to the previous comparative studies concerning the discrimination of taxa based on the characteristics of seasons, in which the application of this algorithm produced the best results (Kubik-Komar et al., 2015). It uses the gain ratio as measure of the diversity of *k* classes (*C*_i_, *i*=1,…,*k*), which is defined as:
(1)
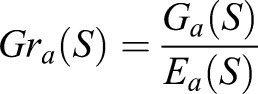
where *E_a_*(*S*) is the entropy (impurity function) of the training set *S* split by the *a* attribute (feature) and *G_a_*(*S*) is the information gain calculated according to the following formulas:
(2)
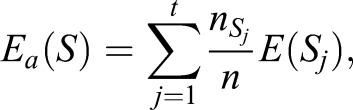

(3)
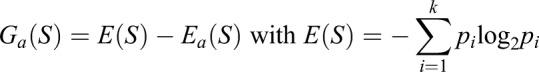


In the Eqns 2 and 3 *S_j_* (*j*=1,…,*t*) are subsets of the training set split by *a*, *p_i_* denotes the probability that the observation is from class *C_i_* estimated as a proportion of the number of observations in *C_i_* to the number of observations in the training set 
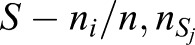
 is the number of observations in *S_j_,* and *n_ij_* is the number of observations from class *C_i_* that appeared in the *S_j_* subset.

For each attribute *a*, the gain ratio is calculated and the one with the maximum value is chosen.

All the classification results and tree diagrams were obtained using the WEKA open source tool for data mining tasks (Hall et al., 2009). Statistical charts were drawn in STATISTICA software (StatSoft, 2011).

Model verification should be done on the testing set whose elements have not participated in building the tree. However, due to the limited number of observations, the models were built and verified using two methods. The first method involved the use of all 225 observations to build the tree. The same observations were used to verify the model, with full awareness that the percentage of correctly classified observations, which is a measure of model classification accuracy, was overstated. The other verification method was fivefold cross-validation (P. Ozer, Data Mining Algorithms for Classification, BSc thesis, Radboud University Nijmegen, 2008). In this case, the observations were assigned to five folds with equal numbers of the individual taxa. Four of these folds were used to build the tree (180 observations, 60 for each taxon), whereas the fifth fold was used to verify it (45 observations, 15 for each taxon). In this way, with each random selection of folds, five trees were built and each of them was created and verified based on a different dataset. This operation was repeated 1000 times in order to make the classification outcome independent of the influence of the random selection.
